# Host–Guest Interactions in the C_59_N^•^⊂[10]CPP Supramolecular Radical

**DOI:** 10.1021/acs.jpcc.4c07474

**Published:** 2025-05-07

**Authors:** Yuri Tanuma, Bastien Anézo, Tilen Knaflič, Jannis Volkmann, Hermann A. Wegner, Ioanna K. Sideri, Nikos Tagmatarchis, Christopher P. Ewels, Denis Arčon

**Affiliations:** † Faculty of Mathematics and Physics, University of Ljubljana, 1000 Ljubljana, Slovenia; ‡ Jožef Stefan Institute, 1000 Ljubljana, Slovenia; § Institut des Matériaux de Nantes Jean Rouxel (IMN), UMR 6502 CNRS, Nantes University, 44322 Nantes, France; ∥ Institute for the Protection of Cultural Heritage of Slovenia, 1000 Ljubljana, Slovenia; ⊥ Institute of Organic Chemistry, Justus Liebig University Giessen, 35392 Giessen, Germany; # Center for Materials Research (ZfM/LaMa), Justus Liebig University Giessen, 35392 Giessen, Germany; ¶ Theoretical and Physical Chemistry Institute, National Hellenic Research Foundation, 11635 Athens, Greece

## Abstract

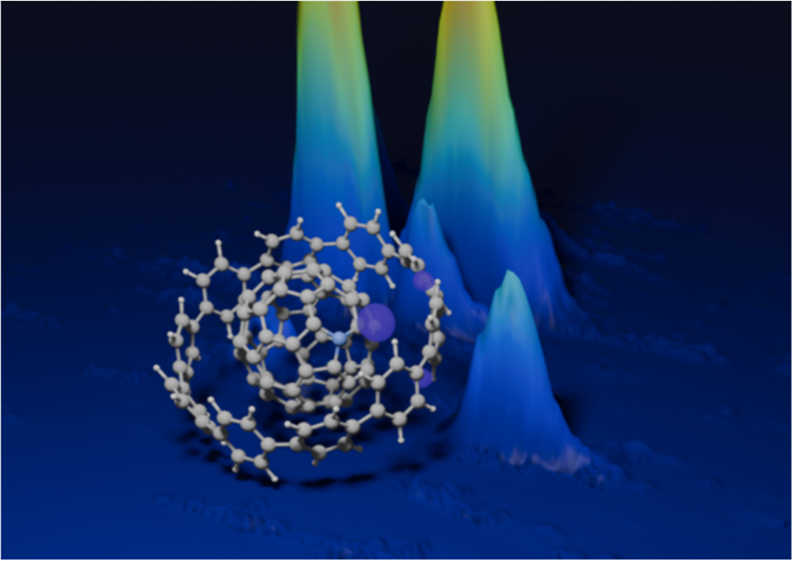

A remarkable stability of a supramolecular radical comprising an azafullerene (C_59_N^•^) radical sterically protected by a [10]cycloparaphenylene ([10]CPP) nanoring, C_59_N^•^⊂[10]CPP, has recently been observed by various experimental probes. In order to investigate the host–guest interaction in these supramolecular complexes, we carried out electron paramagnetic resonance (EPR) measurements, theoretically supported by density functional theory (DFT) calculations. The continuous wave (CW) EPR spectrum shows the presence of two active spin components: C_59_N^•^⊂[10]CPP monomers that can in certain cases polymerize into oligomeric radicals. Two- and three-pulse electron spin echo envelope modulation (ESEEM) measurements allow for determination of experimental hyperfine coupling constants for ^13^C and ^1^H atoms and also show the strong coupling to the ^14^N atom adjacent to the radical C of the azafullerene. These experimental hyperfine coupling constants reasonably agree with those calculated for the DFT optimized supramolecular structures. The results are consistent with a small spin delocalization from the guest (C_59_N^•^) to the host ([10]CPP), thereby explaining weak but non-negligible interaction between them. Our study demonstrates that ESEEM experiments in alliance with DFT computations can offer valuable insights into the radical host–guest structures.

## 1 Introduction

Molecular qubit systems are a simple yet important class of qubits to be potentially used in emerging quantum technology devices.^1^ Molecules with transition metals as their spin-active component are stable, but their use is limited due to the generally large spin–orbit coupling and related short coherence times. On the other hand, spin–orbit coupling is inherently small for organic radicals. Their long-term instability becomes a limiting factor. In the class of light-element-only molecular radicals, fullerene-based radicals may hold interesting qubit properties due to their structural robustness, uniform size, and generally small spin–orbit coupling.

Buckminsterfullerene, C_60_, is a closed shell molecule; but its analogue, the azafullerene C_59_N^•^ is a molecular radical that can be chemically synthesized with high purity.^2^ The C_59_N^•^ radical has an unpaired electron localized mainly on the carbon atom adjacent to the substitutional nitrogen atom. In bulk and solution, it immediately reacts with another C_59_N^•^ radical to form a nonradical dimer (C_59_N)_2_.^2^ With external perturbation, e.g., by exposing dissolved (C_59_N)_2_ to intense laser light (typical λ = 532 nm) or heating up in the solid state, it is possible to break the dimer bond and create C_59_N^•^ radicals.^3−5^ However, after the external perturbation is removed, the radical signal immediately disappears as (C_59_N)_2_ dimers are formed again. The redimerization can be substantially inhibited by entrapping the C_59_N^•^ in a [10]cycloparaphenylene ([10]CPP) ring,^6,7^ and the resultant C_59_N^•^⊂[10]CPP supramolecular radical complex becomes stable on very long time-scales in an inert atmosphere. The C_59_N^•^⊂[10]CPP radical shows strikingly long spin coherence times, as demonstrated by the observation of many Rabi oscillations and measurement of its room-temperature spin–lattice relaxation time *T*_1_ = 210 μm,^6^ which is the longest known among fullerene-based radicals measured at room temperature.

Powder X-ray diffraction (XRD) of C_59_N^•^⊂[10]CPP shows a large unit cell and low symmetry structure but due to the large degree of disorder the structure could not be determined yet.^6^ Very recent scanning tunneling microscopy (STM) studies unambiguously supported that [10]CPP encapsulates C_59_N^•^ to a stable supramolecular structure with the guest C_59_N^•^ sitting in the center of the [10]CPP host. X-ray absorption spectrometry (XAS) demonstrates that the azafullerene in the C_59_N^•^⊂[10]CPP supramolecular complex retains its radical state.^8^ Density functional theory (DFT) computations are consistent with weak π–π coupling between the [10]CPP host and the C_59_N^•^ guest molecular orbitals.^7,8^ This suggests that the spin state of C_59_N^•^⊂[10]CPP is almost identical to that of the C_59_N^•^ radical alone. Moreover, the total energy of the complex has a minimum for the structure where the N atom points toward the [10]CPP ring, which may explain the surprising resilience of C_59_N^•^⊂[10]CPP toward C_59_N^•^ nearest-neighboring redimerization in solution as well as in the solid.^6,7,9^ Yet, the host–guest interaction and the relative orientation of C_59_N^•^ with respect to [10]CPP remain experimentally unconfirmed. This calls for complementary local probe techniques sensitive to the radical state and at the same time capable of probing local structure environment. Here, we report results from detailed electron paramagnetic resonance (EPR) and electron spin echo envelope modulation (ESEEM) analysis supported with the DFT calculations. We find remarkable agreement in EPR parameters between the experiment and theory, thereby supporting the idea that the C_59_N^•^⊂[10]CPP monomer and its oligomer radicals are formed. The supramolecular geometry and interaction between the C_59_N^•^ radical and the [10]CPP nanoring finely tune the hyperfine interactions. These can be directly compared with the experimental values from ESEEM experiments. These data provide constraints for structural refinements of complex radicals with a highly disordered crystal structure that are otherwise not suitable for the XRD analysis.

## 2 Experimental and Theoretical Methods

### 2.1 Sample Preparation

[10]Cycloparaphenylene was synthesized based on a building block strategy using flow chemistry.^10^ Bisazafullerene (C_59_N)_2_ was synthesized following our recent synthetic protocol.^11^ High purity of (C_59_N)_2_ is ensured by the purification procedure followed, which involves (a) column chromatography in *o*-DCB, (b) recrystallization in *o*-DCB/MeOH, and (c) preparative high-performance liquid chromatography (HPLC) with toluene as the mobile phase (concentration of 1 mg/mL, flow rate of 8 mL/min). ^13^C NMR spectroscopy in deuterated *ortho*-dichlorobenzene and UV–vis spectroscopy in dichloromethane revealed the characteristic fingerprint of (C_59_N)_2_, that is in full agreement with the data reported in the literature.^12^ [10]CPP (2.00 equiv) and (C_59_N)_2_ (1.00 equiv) were each dissolved in a minimum amount of CS_2_. Subsequently, the [10]CPP- and the (C_59_N)_2_-containing solutions were combined and stirred for 2.5 days at room temperature. The solvent was removed from the suspension by evaporation yielding a brown solid. The solid was dried for 6 h at 4 × 10^–5^ mbar (room temperature). For the subsequent measurements, the sample was sealed into a Suprasil EPR tube after evacuation to vacuum (*p* < 1 × 10^–5^ mbar) and heated to 550 K to create C_59_N^•^⊂[10]CPP radicals.

### 2.2 DFT Calculations

All the DFT structure optimization was performed using the ORCA package^13−15^ (version 6.0), with the B3LYP functional^16−19^ and 6-31G** basis set^20−25^ with D3 correlation for dispersion forces^26^ and geometrical counterpoise corrections (gCP).^27,28^ The EPR parameter calculations were performed using the EPR-II basis set^29^ for the B3LYP/6-31G**-gCP-D3 geometry-optimized structure. Default settings were used for the other parameters. Molecular images were generated with the Jmol viewer.^30^

### 2.3 CW-EPR Measurement

The Bruker ELEXSYS E-500-4300R spectrometer was used. The measurement was carried out at room temperature with 4.04 mW of microwave power.

Curve fitting of the experimental CW-EPR spectrum was carried out by using the function “pepper” equipped in the MATLAB toolbox “EasySpin” (version 6.0.6).^31^ The experimental system is assumed as a spin system composed of C (98.9% ^12^C, 1.1% ^13^C) and ^14^N radicals. Axial strains are imposed for the *g*-factors (*g*_*x*_ = *g*_*y*_ = *g*_⊥_). Experimentally obtained parameters after the curve fitting are summarized in Table 1.

**Table 1:** Summary of Experimentally Obtained EPR Parameters by the CW-EPR

system	triplet (^14^N)	singlet (^12^C + ^13^C)
*A*_iso_ (MHz)	11.0 ± 0.1	25.0 ± 0.7
*g*_⊥_	2.0007 ± 0.00004	2.0018 ± 0.0004
*g*_*zz*_	2.0035 ± 0.0003	2.0018 ± 0.0008
*g*_iso_	2.0016 ± 0.0003	2.0018 ± 0.0012
Lorentzian fwhm (mT)	0.20 ± 0.01	0.41 ± 0.01
weight	1	3.28 ± 0.49

### 2.4 ESEEM Measurements

Both 2- and 3-pulse ESEEM experiments with the X-band microwave pulse were performed by using the Bruker ELEXSYS E580 spectrometer.

**Figure 1:**
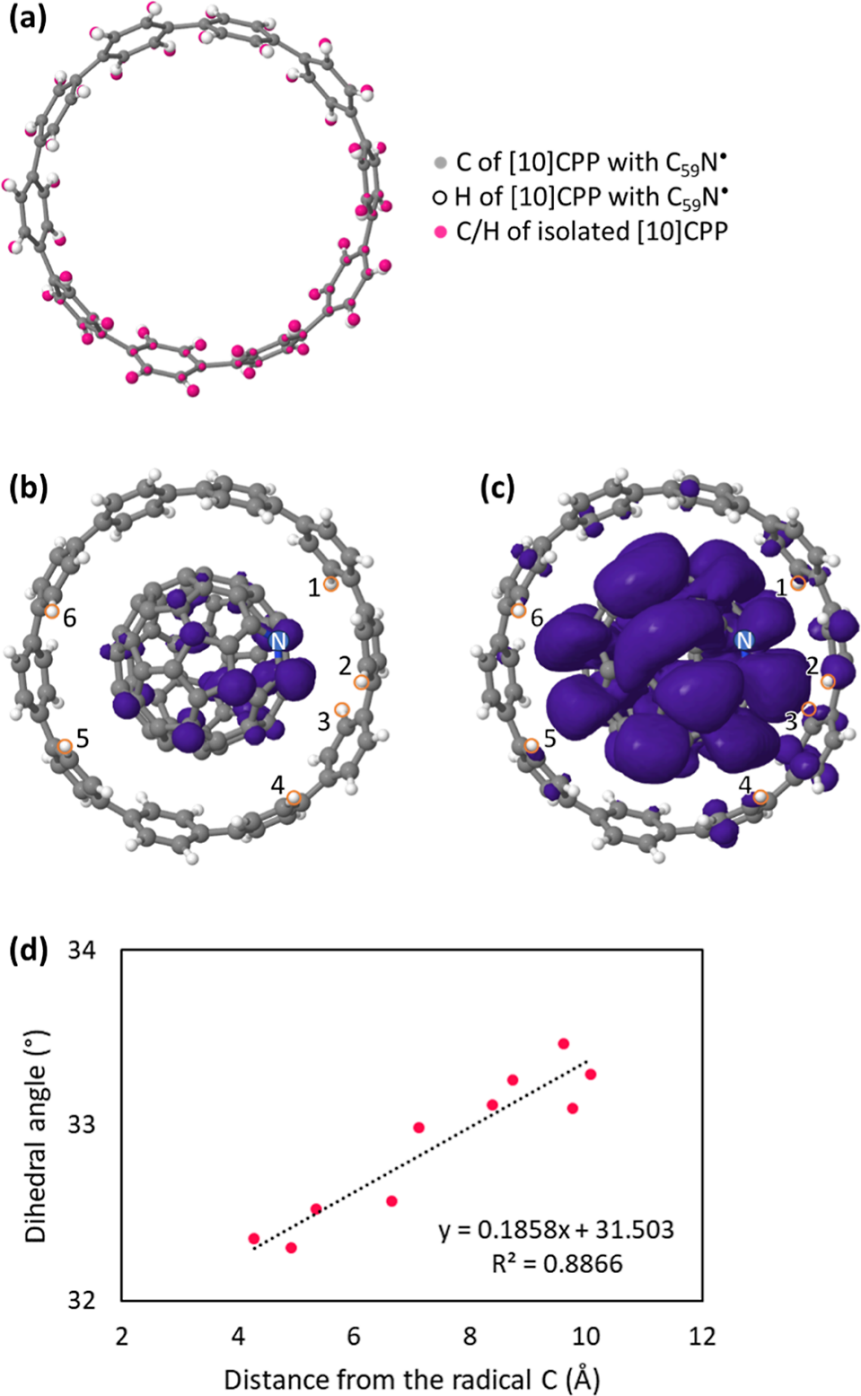
Results of DFT calculations for C_59_N^•^⊂[10]CPP and [10]CPP. (a) Comparison of [10]CPP molecular geometries. Gray and white balls represent carbon and hydrogen atoms of [10]CPP, respectively, with the atomic positions extracted from the geometry-optimized C_59_N^•^⊂[10]CPP molecule. Pink ball shows the atomic positions of carbon and hydrogen atoms in the geometry-optimized isolated [10]CPP. The phenyl dihedral angle is decreased when the [10]CPP accommodates C_59_N^•^. The ring coordinates (to the position of C_59_N^•^) are the same as the C_59_N^•^⊂[10]CPP molecule shown in (b,c). (b,c) Optimized molecular structures of the C_59_N^•^⊂[10]CPP radical with different cutoff values of unpaired electron mapping in purple; (b) 0.002 and (c) 0.00002 *e*/*a*_0_^3^. Nitrogen atom is marked with blue. Labels of orange-circled hydrogen atoms correspond to the hydrogen labels in Table 3. (d) Relation between the dihedral angle of the [10]CPP phenyl rings and the distance from the radical C atom. Black dashed line shows linear approximation.

The 2-pulse ESEEM experiment was carried out for the C_59_N^•^⊂[10]CPP sample at 105 K with a 2-pulse sequence; pulse length τ_p_ = 16, followed by τ_p_ = 16 ns. For the curve fitting, the function “saffron” provided by the EasySpin^31^ toolbox was used. Due to the computational cost, 1 ^13^C (with the natural abundance ratio), 1 N, and 2 different H atoms for system 1 (as the triplet component in the CW spectrum, Figure 4) and 1 ^13^C and 1 H atoms for system 2 (as the singlet component) were taken into account of the curve fitting. Experimentally obtained two-pulse ESEEM parameters after the curve fitting are summarized in Table 2.

**Table 2:** Summary of Experimentally Obtained ESEEM Parameters for (a) Spin System 1 for the Triplet Signal in CW-EPR and (b) Spin System 2 for the Singlet Signal by the 2-Pulse ESEEM Signal

(a) system 1: ^1^H + ^14^N	^1^H	^14^N
*A*_⊥_, *A*_*zz*_ (MHz), *A*_iso_ (MHz)	∓0.9, ±0.9, ∓0.3	8.6, 9.8, 9.0
*g*_⊥_, *g*_*zz*_, *g*_iso_	2.0005, 2.0034, 2.0015	
Lorentzian fwhm (mT)	1.05	
*T*_1_ (μs)	207.3	
*T*_2_ (μs)	5.3	

(b) system 2: ^1^H	^1^H
*A*_⊥_, *A*_*zz*_ (MHz), *A*_iso_ (MHz)	∓0.6, ±0.5, ∓0.3
*g*_⊥_, *g*_*zz*_, *g*_iso_	2.0012, 2.0033, 2.0019
Lorentzian fwhm (mT)	2.23
*T*_1_ (μs)	472.0
*T*_2_ (μs)	20.0

For the 3-pulse ESEEM experiment, we performed a pulse sequence of three π/2 pulses (τ_p_ = 16 ns) at room temperature. Pulse intervals τ and *T* were varied from 160 and 200 ns to 412 and 1220 ns, respectively, both by 4 ns. Obtained spectra were Fourier-transformed along both τ and *T* axes and plotted along ω_1_ and ω_2_ axes, respectively, in the 2D color plot (Figure 6).

## 3 Results and Discussion

### 3.1 Density Functional Theory Computations

#### 3.1.1 [10]CPP Structural Changes during the C_59_N Encapsulation Process

In the family of [*n*]CPP nanorings, neighboring phenyl rings tilt relative to each other.^32^ This dihedral angle is due to steric hindrance caused by the aryl hydrogen atoms.^32^ The dihedral angle becomes smaller with decreasing [*n*]CPP nanoring diameter (i.e., smaller *n*), reflecting the relatively increasing energetic cost of molecular deformation. For an isolated [10]CPP nanoring, our DFT-optimized molecular geometry shows the alternating tilting of phenyl rings by an average dihedral angle of ⟨φ⟩ = 35.3° (Figure 1a, standard deviation is 0.06), consistent with literature values.^33^ When the C_59_N^•^ radical is positioned with its center 4.9 Å above the center of the [10]CPP nanoring, DFT structural optimization spontaneously drives C_59_N^•^ into the center of [10]CPP, i.e., the encapsulation process is barrierless (Figure S1, Data S1). The encapsulation process results in a substantial 2.36 eV release of complexation energy from the two isolated molecules. In this energy minimum structure, the N atom in the C_59_N^•^ is preferentially pointing toward the [10]CPP ring (**structure A**, shown in Figure 1b,c). This supramolecular structure is 0.02 eV more stable compared to an alternative metastable structure with the N atom pointing out of the [10]CPP ring (Figure S2, **structure B**). Experimentally, such an encapsulation process has been recently directly observed by STM for the C_59_N^•^⊂[10]CPP complex on the Au(111) surface using codeposition and thermal annealing protocols. However, in these room-temperature STM measurements the C_59_N^•^ molecular orientation centered in the [10]CPP ring could not be uniquely determined.^8^ In DFT computations, the encapsulation of C_59_N^•^ by [10]CPP slightly decreases the phenyl dihedral angle to an average of ⟨φ⟩ = 32.9° (32.3–33.5°) and increases the standard deviation of the dihedral angles to 0.40 when compared to pristine [10]CPP without C_59_N^•^. Our calculations for C_59_N^•^⊂[10]CPP namely show that the phenyl dihedral angle φ depends on the distance from the radical C site on azafullerene, which demonstrates the [10]CPP symmetry breaking upon C_59_N^•^ encapsulation (Figure 1d). For comparison, the calculated average ⟨φ⟩ for the C_60_⊂[10]CPP structure is 32.95° (32.79–33.29°) with a much smaller standard deviation of 0.18. These comparisons reveal that the decrease in the [10]CPP phenyl dihedral angles is indeed due to the accommodation of fullerene cage inside while attempting to optimize at the same time the π–π contact and the lone pair (lp)–π interaction^34^ between the phenyl ring and the lone pair of the N atom of the host and the guest molecules, respectively.^8^ For more detail, see Annex S1.

#### 3.1.2 C_59_N^•^⊂[10]CPP Spin Distribution

**Figure 2:**
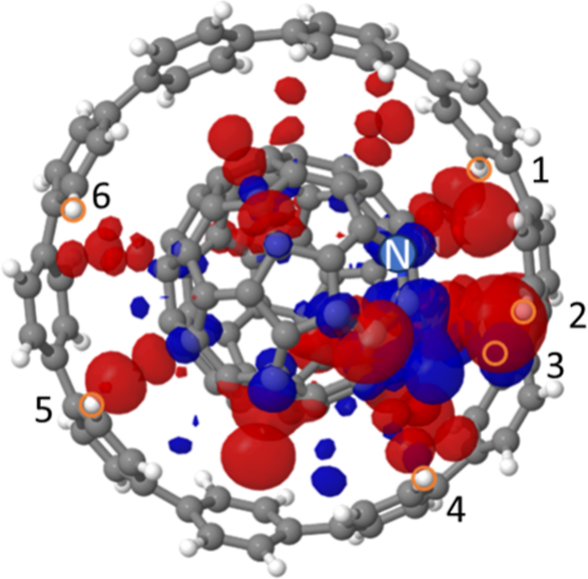
Subtraction of C_59_N^•^ spin density from C_59_N^•^⊂[10]CPP (**structure A**) spin density. Red and blue indicate positive and negative differential spin densities, respectively. The spin distributed in the blue region in C_59_N^•^ moves to the red region when the [10]CPP is added. Isosurface cutoff value is set at 0.002 *e*/*a*_0_^3^. Gray, white, and blue balls represent carbon, hydrogen, and nitrogen atoms, respectively. Labels of orange-circled hydrogen atoms correspond to the hydrogen labels in Table 3.

The DFT computed unpaired electron distribution for an optimized C_59_N^•^⊂[10]CPP geometry is shown in Figure 1b,c. The calculated spin distribution of the C_59_N^•^⊂[10]CPP supramolecular radical is still vastly concentrated on the azafullerene and is very similar to the one of the bare C_59_N^•^ radical without the [10]CPP host^2,6,7^—the majority of unpaired electron density is localized on the carbon atom adjacent to the nitrogen atom of the C_59_N^•^ cage (Figure 1b). However, a main difference from the bare C_59_N^•^ radical is that for **structure A** of the C_59_N^•^⊂[10]CPP complex a tiny amount of spin density shifts to the [10]CPP nanoring (Figure 1c). To visualize this transfer, we in Figure 2 show a difference between the spin density on parent C_59_N^•^ and one of the C_59_N^•^⊂[10]CPP complexes. Mulliken spin population analysis shows that 0.5% of the total spin is after C_59_N^•^ encapsulation distributed on the phenyl rings of the [10]CPP host. More specifically, the same analysis reveals that the largest amount of the transferred spin on the [10]CPP amounts to ≈24.5% for a C atom, which is closest to the radical C atom of C_59_N^•^. This tiny spin redistribution on the [10]CPP therefore suggests the existence of weak hybridization between their respective molecular orbitals. Interestingly, in the case of **structure B**, ≈0.7% of the total spin is transferred to the [10]CPP (Figure S3) thus implying similar weak hybridization effects. We note that the spin distribution for this model structure is similar to the optimized C_59_N^•^⊂[10]CPP **structure A** with minute spin density on the [10]CPP host (Table S1 and Figure S2b). From this perspective, the **structures A** and **B** are not distinguishable since the difference in the transferred spin density to [10]CPP is still very small.

#### 3.1.3 DFT-Calculated EPR Parameters

DFT calculations on the relaxed C_59_N^•^⊂[10]CPP geometries allow us to calculate their EPR parameters. For the optimized **structure A** where the N atom of the azafullerene points toward one of the phenyl rings of the host [10]CPP nanoring, the calculated radical *g*-factor values are *g*_iso_ = 2.0011, with eigenvalues of *g*_1_ = 2.0007, *g*_2_ = 2.0009, and *g*_3_ = 2.0017. These *g*-factor values are nearly identical to *g*_iso_ = 2.0011 (*g*_1_ = 2.0007, *g*_2_ = 2.0009, and *g*_3_ = 2.0019) for the bare C_59_N^•^ radical without the [10]CPP ring and reflect remarkable radical stability upon the encapsulation. For comparison, we also calculated *g*-values for the C_59_N^•^⊂[10]CPP **structure B** with the N pointing out of the [10]CPP ring (Figure S2) and obtained *g*_iso_ = 2.0011 (*g*_1_ = 2.0006, *g*_2_ = 2.0009, and *g*_3_ = 2.0019). The similarity of the calculated *g*-factors for the three different model radical structures is due to the very small spin transfer between the guest C_59_N^•^ radical and the host [10]CPP and it would be therefore experimentally extremely difficult to distinguish them.

#### 3.1.4 Spin Density on H Sites of the [10]CPP Nanoring

An alternative route to test different competing C_59_N^•^⊂[10]CPP structures would be to explore the nuclear isotropic hyperfine coupling parameters (*A*_iso_) as a magnifying glass for the minute spin density transfer to the host [10]CPP. The DFT-calculated *A*_iso_ values for ^1^H atoms of [10]CPP host in the geometry-optimized **structure A** with absolute values larger than 0.1 MHz are summarized in Table 3. Only 6 out of 40 ^1^H atoms satisfy this criterion. These small *A*_iso_ values are consistent with the weak hybridization between the C_59_N^•^ and [10]CPP and suggest that the main interaction between the unpaired spin density of C_59_N^•^⊂[10]CPP and these ^1^H atoms are of a dipolar nature. We note that one of the ^1^H atoms with largest *A*_iso_ is surprisingly located not at the close vicinity of the radical C atom but at the almost opposite side of the cage (Figure 1c, ^1^H atom labeled 5). On a C_59_N^•^, the spin is not exclusively “localized” on the C next to the N, but small amount of spin is distributed also on other C atoms as seen in Figure 1b. Indeed, this observation is entirely consistent with the spin density subtraction of C_59_N^•^ from C_59_N^•^⊂[10]CPP (Figure 2), in which the H atoms with the largest *A*_iso_ values (labeled 1 ∼ 6 in Figure 2) are readily recognized close to red-colored regions. While these small *A*_iso_ may still allow us to uncover fine structural details of the C_59_N^•^⊂[10]CPP complex, we note that the activation energy to rotate C_59_N^•^ within the [10]CPP ring keeping the N atom beneath the ring edge is less than 0.2 eV.^7^ Also, the C_59_N^•^ would spin around in the [10]CPP at room temperature due to the small energy difference between **structure A** and **B**. Therefore, under ambient conditions the C_59_N^•^ radical will likely rapidly rotate inside the [10]CPP nanoring, and the experimentally measurable distance from the radical to the given H atoms may be averaged on the time-scale of EPR measurements.

**Table 3:**
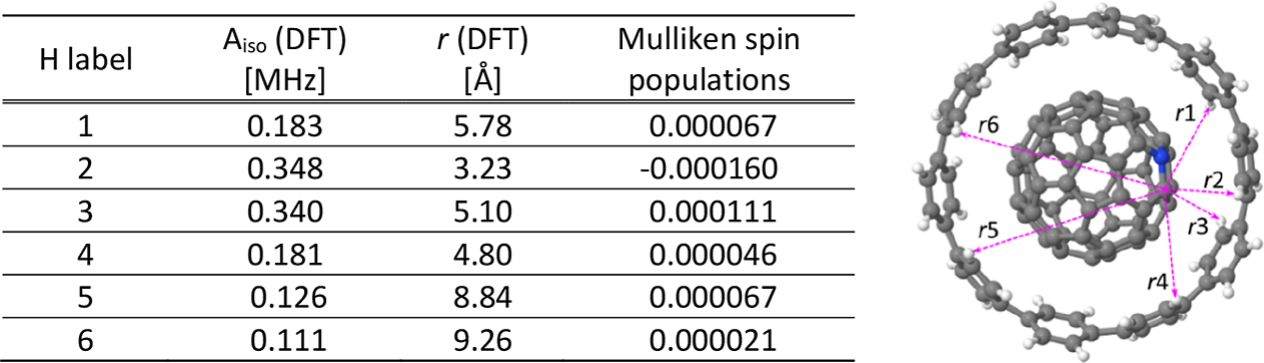
Summary of *A*_iso_^H^ Values for **Structure A** with Absolute Values Larger than 0.1 MHz in [10]CPP and Its C–H Distance (*r*) from the Radical Carbon Atom of C_59_N^•^, Calculated by DFT. Pink Dashed Arrows in the Molecular Structure in the Right Panel Show *r* with H Label in the Table

#### 3.1.5 Spin Density on N and C Sites of the C_59_N^•^

The DFT isotropic hyperfine coupling values for the ^14^N and the radical ^13^C atoms of C_59_N^•^ component for the 3 radical structures (C_59_N^•^ without [10]CPP, **structure A**, and **structure B**) are summarized in Table S1. Given the very small difference in the spin dentistry on C_59_N^•^ for the considered model structures, it is not surprising that ^14^N and ^13^C values of hyperfine coupling are very similar, including the hyperfine anisotropy. The similarity of all DFT calculated EPR parameters for the 3 radical structures renders these structures experimentally almost indistinguishable. Overall, we can conclude that for the various competing C_59_N^•^⊂[10]CPP supramolecular structures, the influence of the [10]CPP nanoring on the spin parameters, which are measurable in continuous wave (CW) EPR experiments, is small enough to neglect it in the further discussion. The largest differences appear due to the changes in phenyl dihedral angles upon complexation, which could be probed by exploring radical-^1^H dipolar interaction.

#### 3.1.6 DFT for Trimer as Oligomer Radicals

**Figure 3:**
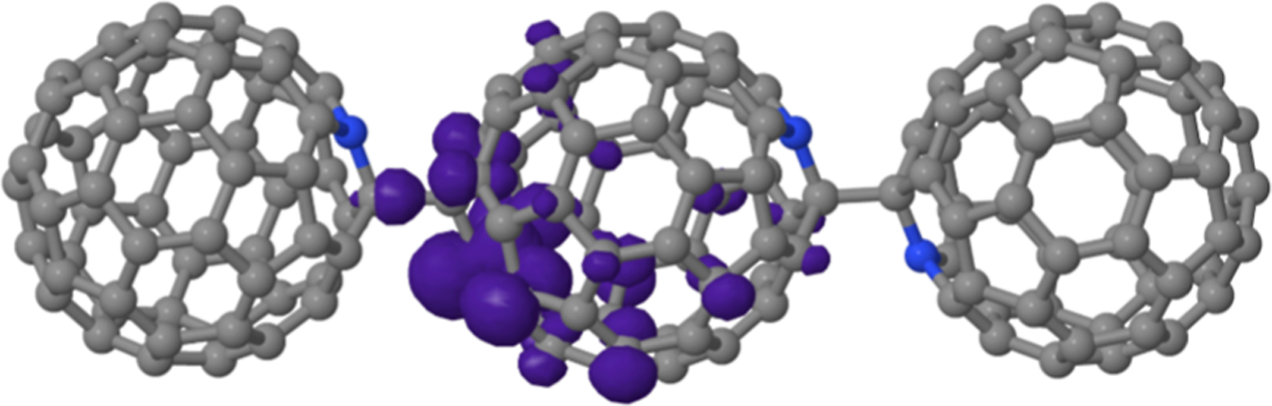
Molecular structure of an azafullerene oligomer radical; trimer radical. Gray, white, and blue balls represent carbon, hydrogen, and nitrogen atoms, respectively. Purple bubbles show spin distribution with an isosurface cutoff value set at 0.002 *e*/*a*_0_^3^. [10]CPP rings are omitted for the visibility of the spin distribution and the calculation cost.

Finally, we stress that in C_59_N^•^⊂[10]CPP solids another possible radical structure may emerge.^6^ This structure is obtained in solids when (1) C_59_N^•^⊂[10]CPP is generated from the parent dimer by, e.g., thermolysis process, (2) the created C_59_N^•^ rotates in the lattice until (3) the radical carbon attaches to the rear of an azafullerene in a nearest neighboring EPR-silent [10]CPP⊃(C_59_N)_2_⊂[10]CPP complex.^6^ This process leads to an unusual trimer structure displayed in Figure 3. In the case of such oligomer supramolecular radicals, the interfullerene bonding eliminates the radical on the initial azafullerene but creates one on the rear of the azafullerene dimer it is bonding to. In this way the spin density is effectively transferred to C sites on the rear of the central azafullerene (Figure 3).^6^ As a result, the local bonding and radical distribution are very different from those of the azafullerene monomer. This is clearly reflected in DFT-calculated isotropic hyperfine coupling constants for the model C_59_N^•^-(C_59_N)_2_ trimer radical. The most striking difference is a very small value for the ^14^N isotropic hyperfine coupling constant, which amounts to only 1.91 MHz.^6^ The characteristic ^14^N splitting of the EPR spectrum, which is a fingerprint of the C_59_N^•^ radical, should thus be absent for such a trimer supramolecular radical. The two C sites with the largest values of 78.86 and 77.41 MHz are at the intercage C of the left cage and adjacent to the intercage C of the central cage, respectively, as shown in Figure 3.^6^

### 3.2 Continuous Wave X-Band EPR Spectrum

**Figure 4:**
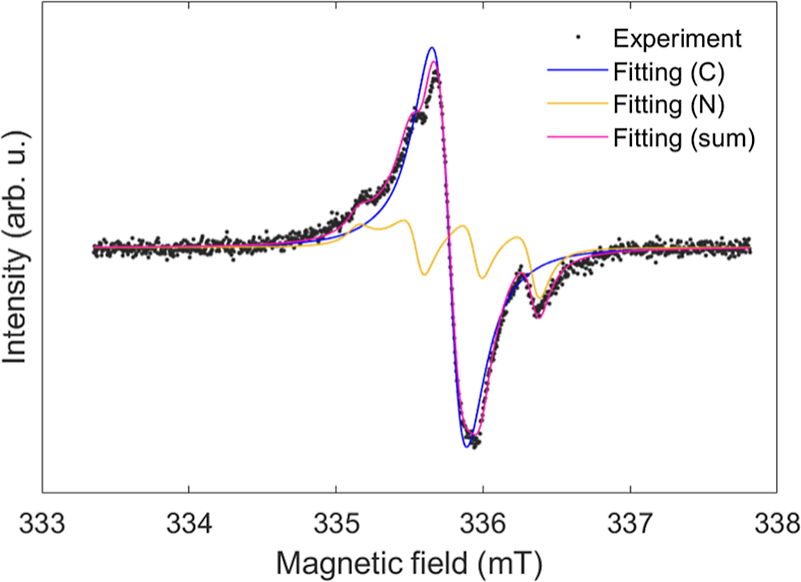
Room-temperature CW X-band EPR spectrum of thermally treated [10]CPP⊃(C_59_N)_2_⊂[10]CPP powder (black dots). The curve fitting simulation (pink line) assumes the coexistence of a C_59_N^•^⊂[10]CPP radical showing characteristic ^14^N hyperfine splitting (yellow line) and another singlet component (blue line) that is ascribed to the formation of trimer or higher oligomers.

A room-temperature CW X-band EPR spectrum of the C_59_N^•^⊂[10]CPP powder is a superposition of two overlapping signals with triplet and singlet features (Figure 4). Based on the above DFT calculations for the two different model C_59_N^•^⊂[10]CPP structures, the Zeeman and hyperfine terms for ^1^H atoms are always very small and thus can be neglected in the spectral simulation (fitting details can be found in the Methods section). Since the DFT calculations generally show *g*_*x*_ ≈ *g*_*y*_, we assume in our simulations that the *g*-factors have axial symmetry (*g*_*x*_ = *g*_*y*_ = *g*_⊥_). Components of nuclear hyperfine coupling tensors (*A*) calculated by DFT also indicate their axial symmetry. However, due to a limited signal-to-noise ratio in our measurements, inclusion of hyperfine coupling tensor asymmetry does not improve the quality of the fits compared to the fit based just on isotropic hyperfine coupling interactions. Therefore, we report in Figure 4 and Table 1 the line shape fit and the list of fitting parameters without considering the anisotropy of hyperfine coupling interactions. The triplet features of the EPR spectrum (Figure 4), which match the EPR spectra reported in the literature for the C_59_N^•^⊂[10]CPP powder samples,^6^ are due to the hyperfine coupling to the nitrogen which is adjacent to the radical carbon atom. This hyperfine coupling has been taken in the past as a fingerprint of C_59_N^•^⊂[10]CPP created in [10]CPP⊃(C_59_N)_2_⊂[10]CPP either by photo- or thermolysis.^6,7^ The powder X-band EPR line shape fitting for this triplet component converges to an axially anisotropic *g*-factor with components *g*_⊥_ = 2.0007 and *g*_*zz*_ = 2.0035 (*g*_iso_ = 2.0016) and the dominant ^14^N hyperfine coupling with *A*_iso_^N^ = 11.0 MHz. This *A*_iso_^N^ value is, however, somewhat higher than the 10.1 MHz reported in ref (6). In this study, we refine our fitting model by incorporating *g*-factor anisotropy, which was not considered previously in ref (6). That work employed a simplified model assuming isotropic *g*-factor and isotropic hyperfine coupling, with line shape details absorbed into a line-broadening parameter. The additional broadening introduced by *g*-factor anisotropy in this study influences the extracted parameters, leading to a more accurate determination of the *A*_iso_^N^. The dominance of ^14^N hyperfine coupling does not allow us to extract weaker hyperfine coupling to ^13^C nuclear moments for the triplet component. As the coexistence of oligomer radicals (e.g., Figure 3) with the monomer radicals has been reported previously,^6^ we associate the singlet component with this type of radical species. For the singlet feature, we therefore added in the EPR spectrum simulation a component with a hyperfine coupling to the C site (we took the ^13^C concentration in its natural abundance of 1.1%) and neglected any hyperfine coupling to nitrogen. The fitting simulation for this component yields the hyperfine coupling constant *A*_iso_^C^ = 25.0 MHz. The derived parameters for the spin Hamiltonian of the C_59_N^•^⊂[10]CPP supramolecular radical are remarkably consistent with the theoretical values calculated here by the DFT calculations. Therefore, here it is simply fitted as a component having a Lorentzian line shape and an isotropic *g*-factor of 2.0018. The fitting with a simple Lorentzian line shape without explicitly involving ^13^C hyperfine coupling, thus incorporating unresolved ^13^C hyperfine interactions within the simulated line width, we obtain reduced χ^2^ = 1.997. When ^13^C hyperfine interactions are explicitly included, χ^2^ is marginally reduced to χ^2^ = 1.941. The similarity in χ^2^ for the two approaches probably arises from the fact that the electron wave function spreads over many C sites on the fullerene cage. We note that we could not carry out DFT calculations of EPR parameters for the trimer and oligomers due to the very large calculation cost.

### 3.3 Electron Spin Echo Envelope Modulation Simulations and Assignments

**Figure 5:**
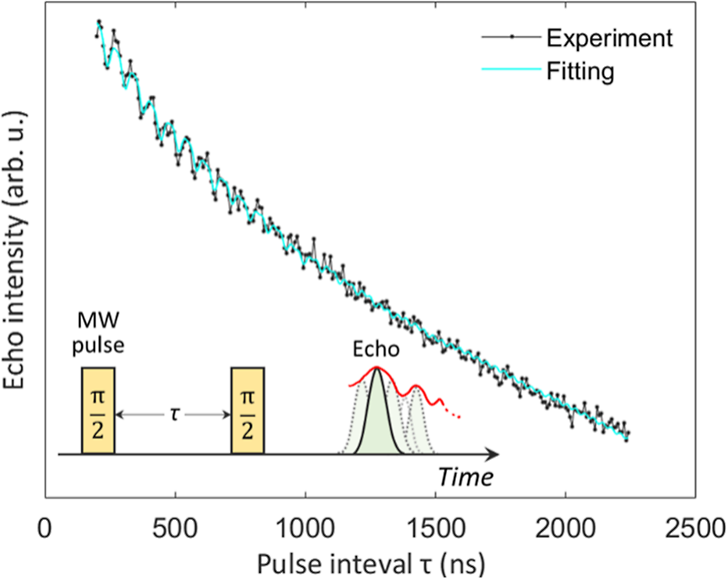
2-Pulse ESEEM spectrum of C_59_N^•^⊂[10]CPP. Black points and green line represent experimental and fitting results, respectively. The schematic diagram of the 2-pulse sequence along the time-axis is shown in inset.

Pulsed EPR techniques enable us to determine weaker spin interactions, which cannot be otherwise resolved in the CW-EPR experiments. Electron spin echo envelope modulation (ESEEM) experiments are particularly powerful for the resolution of hyperfine coupling interactions. In a 2-pulse ESEEM experiment comprising two π/2 pulses separated by a variable delay time τ (the solid echo pulse sequence, inset in Figure 5), the generated spin echo signal intensity decays exponentially with τ and the echo decay is modulated by weak interactions with neighboring nuclei.^35^ Indeed, in a 2-pulse ESEEM measurement carried out for the C_59_N^•^⊂[10]CPP powder sample at a temperature *T* = 105 K, we observe a clear echo-signal-intensity modulation with the main modulation period of Δ*t* = 70.7 ns being superimposed on the exponential decay (Figure 5). Mathematical explanation for the ESEEM signal can be found in standard textbooks, e.g., in ref (38), while the main expressions are also summarized in Annex S2.

The echo signal decay fitting requires two components with different spin–spin relaxation times, *T*_2_, converging to values of 5.3 and 20.0 μs, respectively. We note that the extracted *T*_2_ values are comparable or even longer to the corresponding values at room temperature (631 ns)^6^ or when compared to the similar N@C_60_ system studied at 190 K (5 μs).^36^ This is an important property should these radical centers be explored as qubits in the future. Importantly, the two-component echo decay also mirrors two components identified in the CW-EPR measurements (Figure 4). Therefore, we associate them with the C_59_N^•^⊂[10]CPP monomers and oligomers, respectively.

For each of the two components, we thus proceed with the 2-pulse ESEEM signal *s*_*i*_(τ) analysis using
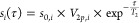
1where *s*_0,*i*_ is the initial amplitude of the echo-signal-intensity for a given component indexed as *i* = 1 (monomers) and *i* = 2 (oligomers). *V*_2*p*,*i*_ is the ESEEM modulation (eq S1). The period of ESEEM signal modulation Δ*t* = 70.7 ns suggests that the ESEEM modulation of the signal has a frequency of ν = 1/Δ*t* = 14.14 MHz, which is close to the ^1^H Larmor frequency (14.60 MHz) in the experimental magnetic field of *B*_0_ = 342.8 mT. The observed modulation thus implies weak dipolar coupling to the nearby protons (Figure 5). This agrees well with our DFT computations also showing extremely small ^1^H contact-hyperfine coupling constants (Table 3). Hence, it is reasonable to assume that the dominant interaction between the C_59_N^•^ radical center and the hydrogen nucleus of the encapsulating [10]CPP nanoring at a distance *r* is of dipolar nature. In such a case, the hyperfine coupling with a target nucleus is weak (*B* ≪ ω_L_ ≈ ω_α_ ≈ ω_β_), and one can explore the dipolar interaction to explicitly express the ESEEM modulation depth *k* as^35^
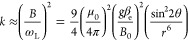
2where β_e_ is the Bohr magneton. Because of the powder nature of the studied sample, the expressions giving the ESEEM signal listed in the eqs 1, 2, and S1–S3 have to be averaged over all possible angles θ and thus the angular averaging gives ⟨sin^2^ 2θ⟩ = 1/2. Finally, combining eqs 2 and S3, the distance *r* between the electron spin and the hydrogen nuclear spin can be estimated. We carried out Fourier transformation of the raw spectrum after subtraction of the exponential baseline (Figure S4). We find the strongest peak at 14.5 MHz, which is the Larmor frequency of hydrogen nuclei. Signals centered at nitrogen and carbon Larmor frequencies are also found at 1.1 and 3.5 MHz, while the peaks at 2.2 and 29.1 MHz are the double nitrogen and hydrogen Larmor frequencies, respectively. We attribute the small peak at 35.3 MHz to ω_+_ = ω_α_^C^ + ω_β_^H^, where ω^C^ is the radical carbon. Weak peaks between 5 and 12 MHz are considered to be ^13^C signals from the different carbon sites (Figure S4 inset). The same weak signals are also observed in 3-pulse ESEEM experiment, which will be shown in the next section. Fitting of echo-decay intensity with the two *s*_*i*_(τ) components, each with its own *T*_2_ and ESEEM modulation *V*_2p,*i*_, is carried out focusing on the clearly resolved modulations at early times τ (Figure 5)—see the Method section for more detail and summary of the fitting. The fit shown in Figure 5 yields coupling to hydrogen atoms at distance *r*(H) = 6.7 for the C_59_N^•^⊂[10]CPP monomer component and *r*(H) = 7.1 Å for the oligomer azafullerenyl radicals. The agreement is even better considering that the experimentally measurable *r* is averaged (6.2 Å for **structure A** (Table 3), and 7.1 Å for **structure B** (Table S2)) due to the C_59_N^•^ cage rotation in the [10]CPP nanoring. Despite several approximations used in this analysis, i.e., we are unable to resolve weak Fermi contact interactions with the ring H atoms, we find that the estimated spin-H distances are consistent with the DFT-calculated main radical C–H distances for the tested C_59_N^•^⊂[10]CPP models that predict some of the spin density to be transferred to [10]CPP rings. Yet, some of the other finer hyperfine couplings that could test our model structures still need to be determined. In particular, hyperfine coupling to ^13^C could not be determined from the 2-pulse ESEEM experiments because of low natural abundance of ^13^C isotope and due to the distribution of ^13^C hyperfine couplings as the electron density spreads over several C sites on the fullerene cage.

**Figure 6:**
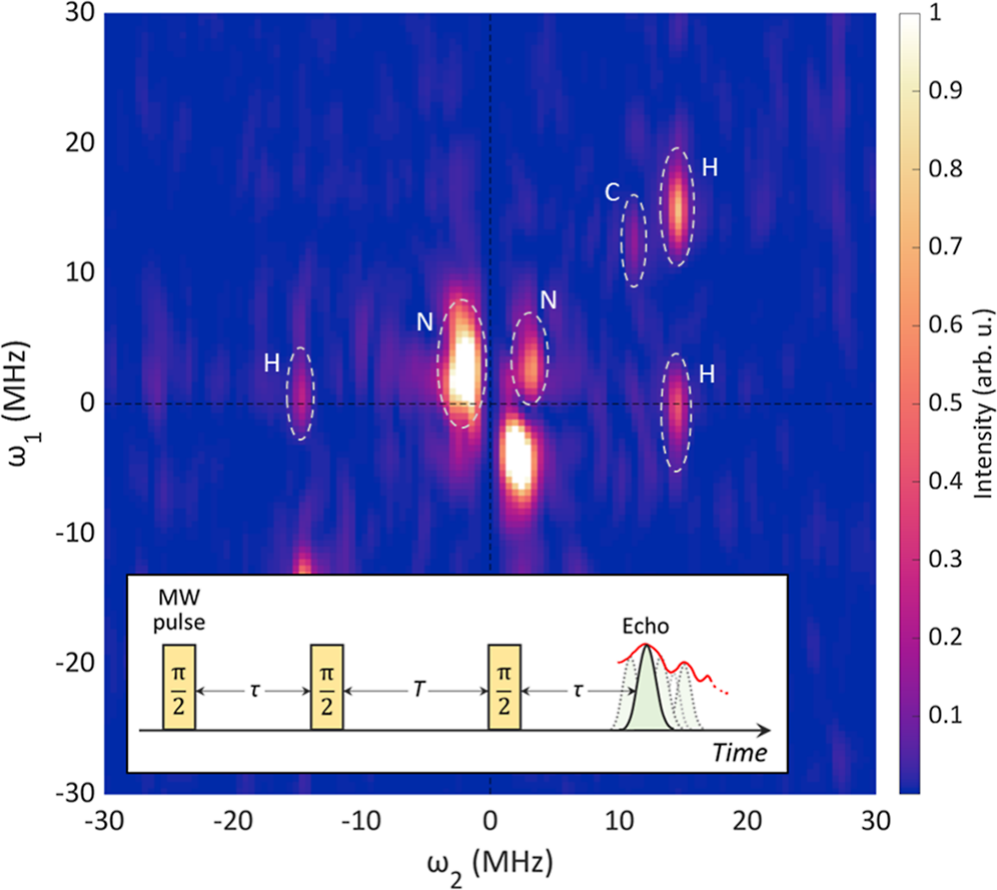
2D 3-Pulse ESEEM spectra after Fourier transformation. ω_1_ and ω_2_ are nuclear frequency along τ and *T* axes, respectively. Intensity is shown by color. Inset shows schematic diagram of the 3-pulse ESEEM pulse sequence. τ and *T* are variable pulse intervals after the first and second microwave pulses, respectively. Intensity is shown by color, with a cutoff value of 1. The color map by Kovesi, P. “Good Colour Maps: How to Design Them.” 10.48550/arXiv.1509.03700 (ref (38)), released under CC BY 4.0 (https://creativecommons.org/licenses/by/4.0/) is used.

To address other more “silent” hyperfine couplings, we therefore next carried out 3-pulse ESEEM measurements.^37^ We used a conventional pulse sequence comprising three π/2 pulses with two-pulse delays τ and *T* (Figure 6 inset). The advantage of these experiments is the spin coherences between the second and the third pulse decay with a longer nuclear relaxation time *T*_M_^*n*^, thus significantly improving the experimental resolution.^35^ Another advantage of the 3-pulse ESEEM is fewer frequencies to consider as the modulation formula can be written as
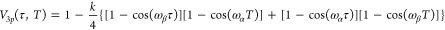
3

For more details of the calculation, see Annex S3. We plotted 3-pulse ESEEM signal intensity along the two axes τ (= ω_1_ axis) and *T* (= ω_2_ axis), and ω_α_ and ω_β_ are derived by the two-dimensional Fourier transformation of the modulation signal. Experimentally obtained two-dimensional (2D) plot of the 3-pulse ESEEM signal at room temperature after the Fourier transformation is shown in Figure 6 and comprises several clearly recognized peaks marked with dashed lines.

As we observed the clearly visible singlet signal in the CW-EPR spectrum, we consider that the 2D 3-pulse spectrum contains 2 spectral components as well. First, the 3-pulse ESEEM signal at (ω_2_, ω_1_) = (14.6 ± 0.7 MHz, 14.6 ± 2.0 MHz) and the axial peaks (14.6 ± 0.2 MHz, 0 ± 1.9 MHz) and (−14.6 ± 0.5 MHz, 0 ± 1.9 MHz) along the ω_1_ axis are immediately assigned as hydrogen-origin peaks. They corroborate the 2-pulse ESEEM experiment presented in Figure 5. The splitting of ω_α_ and ω_β_ is very small, as anticipated from our DFT calculations of weak ^1^H hyperfine couplings even to the closest H1 and H2 sites (Table 3), therefore, ω_α_ and ω_β_ are not separated and we cannot distinguish (ω_α_, ω_α_) and (ω_β_, ω_β_). Yet, using these two ω_α,β_ values under an assumption that the hyperfine coupling constant for H is small enough to assume it is nearly isotropic, the experimental isotropic hyperfine coupling constant for the ^1^H is 0.14 MHz in quantitative agreement with DFT computations. Eq 3 predicts that other ESEEM peaks at (ω_α_, |ω_+_|) = (14.6 MHz, 29 MHz), (ω_β_, |ω_–_|) = (14.6 MHz, 0), and (−ω_α_, |ω_–_|) = (−14.6 MHz, 0) should be detected too.^35^ The two peaks at ω_1_ = |ω_–_| are indeed detected but the other peak at ω_1_ = |ω_+_| peak is not clearly visible because the signal sensitivity decreases at higher frequency and thus the signal tends to be broadened and weakened.

Using the same analysis approach also for ^13^C ESEEM signals, we find that the (ω_2_, ω_1_) = (11.2 ± 0.2 MHz, 12.2 ± 1.7 MHz) peak corresponds to the (ω_α_, ω_α_) spot by assuming that its hyperfine coupling constant is negative (≈−30 MHz), which is what we calculated by the DFT method (Table S1 and ref (6)). From this ω_α_, experimental isotropic hyperfine coupling constant for ^13^C can be calculated as −29.7 MHz, which matches 2-pulse ESEEM and DFT calculations for the radical carbon site on C_59_N^•^. We stress that besides the main ^13^C peak at the frequency of (ω_2_, ω_1_) = (11.2 MHz, 12.2 MHz), there are several weaker peaks in this frequency range. These weaker peaks are however difficult to clearly resolve. Nevertheless, the extracted ^13^C frequencies fall in the correct range for the C sites with the largest hyperfine coupling constants, which supports our peak assignment.

Finally, ^14^N signals are found at (ω_2_, ω_1_) = (3.4 ± 0.5 MHz, 2.9 ± 1.9 MHz) and (−1.5 ± 1.0 MHz, 2.4 ± 3.7 MHz) which are assigned to (ω_β_, |ω_–_|) and (−ω_α_, |ω_–_|) peaks, respectively. Both peaks are close to origin due to the small Larmor frequency ω_L_^N^ = 1.05 MHz and are broadened possibly due to overlapping by some weak ^13^C peaks and multiple orientation of N. The strong ^14^N signal intensity is consistent with the DFT prediction that the electron spin is mainly localized next to the N atom. The experimental *A*_iso_^N^ value measured from the ω_β_ of ^14^N peaks is 8.9 MHz, which is again in a range consistent with DFT calculations.

Thereby, CW-EPR and 2- and 3-pulse ESEEM experiments are fully compliant with the spin distribution presented in Figure 2. Note that the discussion of 3-pulse ESEEM data is the following: (i) we start from our DFT calculations and (ii) then from these values we get the range for the positions of ^1^H peaks in 3-pulse ESEEM experiments. Thus, the experimental and DFT data do not disagree. Although present experimental results lack sufficient resolution to unambiguously confirm our conclusions, we find consistency in the general agreement with the combined CW, 2-pulse and 3-pulse ESEEM measurements. However, the difference between 2 competing structures *A* and *B* is extremely small and calls for more precise techniques, such as HYSCORE.^39^

## 4 Conclusions

We theoretically and experimentally explored the host–guest interaction in the radical supramolecular structure of C_59_N^•^⊂[10]CPP and found that the [10]CPP ring slightly deforms its phenyl chain when it accommodates C_59_N^•^ and becomes a concave–convex complex radical. The two conformations of C_59_N^•^⊂[10]CPP show very little orbital hybridization and therefore the radical spin density remains almost completely localized on azafullerene in agreement with the strong nitrogen ESEEM signal. This explains the remarkable C_59_N^•^⊂[10]CPP radical stability regularly reported in the literature. The modeled radical spin-^1^H distance is determined to be ∼6–7 Å, which is consistent with (i) the DFT results for the geometrically relaxed structure in which radical carbon points toward the [10]CPP ring and (ii) the hydrogen ESEEM signal. The weak host–guest interaction is also responsible for the remarkably long relaxation time, *T*_2_ = 5.3 μs (at 105 K), which is important for any molecular qubit state manipulation. Interestingly, we also confirm the partial transformation of C_59_N^•^⊂[10]CPP into radical oligomers due to bonding to nonmagnetic dimer units. The present study thus demonstrates that the concept of radical encapsulation works well for the studied C_59_N^•^⊂[10]CPP supramolecular structures and throws additional light on the origin of radical stability, as well as their inherently long coherence times. The study also exposes possible structural configurations for the C_59_N^•^⊂[10]CPP. Although the resolution of the ESEEM signals is in our case not sufficient to discriminate between 2 competing **structures A** and **B**, it is nevertheless a valuable method for the determination of spin densities even in solids with high degree of disorder when coupled with DFT.

## Supporting Information Available

Energy vs DFT iteration for the geometry optimization of C_59_N^•^ and [10]CPP molecules closely positioned to each other, DFT-calculated molecular structure and spin distribution of C_59_N^•^⊂[10]CPP (**structure B**), subtraction of C_59_N^•^ spin density from C_59_N^•^⊂[10]CPP (**structure B**) spin density, Fourier transformation of raw experimental 2-pulse ESEEM, summary of difference in DFT-calculated parameters among C_59_N^•^ without [10]CPP and C_59_N^•^⊂[10]CPP complex **structure A** and **B**, summary of *A*_iso_^H^ values for **structure B** with absolute values larger than 0.1 MHz, [10]CPP ring distortion and lp–π interaction, mathematical explanation of ESEEM modulation in the 2-pulse sequence, and calculation of experimental hyperfine coupling constants from 2D plot of 3-pulse ESEEM (PDF)
.xyz data of the encapsulation process of C_59_N^•^ in the [10]CPP ring (XYZ)

## Supplementary Material

jp4c07474_si_001.pdf

jp4c07474_si_002.xyz
