# Supplementary material for: Host–Guest Interactions in the C59N•⊂[10]CPP Supramolecular Radical
Source: J Phys Chem C Nanomater Interfaces. 2025 May 7;129(19):9057–65. doi: 10.1021/acs.jpcc.4c07474 (PMC12086853; doi:10.1021/acs.jpcc.4c07474)
Supplement: Supplementary file 1 — jp4c07474_si_001.pdf [file jp4c07474_si_001.pdf]

Host-guest interactions in the $C_{59}N^{\bullet} \subset [10]CPP$ supramolecular radical

Yuri Tanuma[1,2], Bastien Anézo[2,3], Tilen Knaflič[2,4], Jannis Volkmann[5,6], Hermann A. Wegner[5,6], Ioanna K. Sideri[7], Nikos Tagmatarchis[7], Christopher P. Ewels[3], and Denis Arčon[1,2]*

[1] Faculty of Mathematics and Physics, University of Ljubljana, 1000 Ljubljana, Slovenia.

[2] Jožef Stefan Institute, 1000 Ljubljana, Slovenia.

[3] Institut des Matériaux de Nantes Jean Rouxel (IMN), UMR 6502 CNRS, Nantes University, 44322 Nantes, France.

[4] Institute for the Protection of Cultural Heritage of Slovenia, 1000 Ljubljana, Slovenia

[5] Institute of Organic Chemistry, Justus Liebig University Giessen, 35392 Giessen, Germany.

[6] Center for Materials research (ZfM/LaMa), Justus Liebig University Giessen, 35392 Giessen, Germany.

[7] Theoretical and Physical Chemistry Institute, National Hellenic Research Foundation, 11635 Athens, Greece.

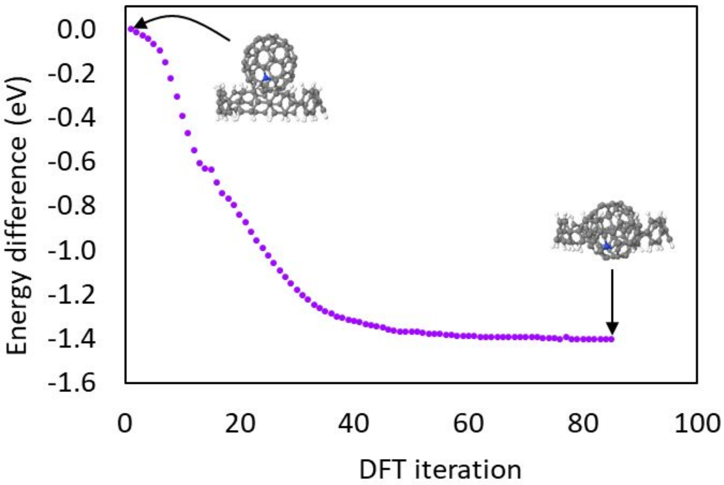

Figure S1. Energy vs DFT iteration for the geometry optimisation of $C_{59}N^{\bullet}$ and [10]CPP molecules closely positioned to each other. Vertical axis shows difference of total energy from the initial structure. 85 iterations were carried out in total and the initial and final molecular structures are shown by black arrows at the data points of 1st and 85th iterations, respectively. Any local minima do not appear (xyz data of this optimisation sequence is shown in Data S1). Thus, the complexation process is barrierless.

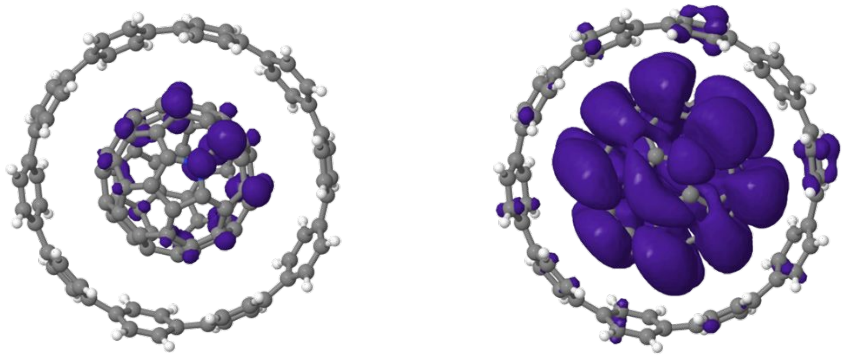

(a) (b)

Figure S2. Calculated molecular structure of $C_{59}N^{\bullet}\subset$[10]CPP with N atom pointing out of [10]CPP ring. Grey, white and blue balls represent carbon, hydrogen and nitrogen atoms, respectively. Unpaired electron distribution is shown by purple bubble plot with a cut-off value of (a) 0.002 and (b) 0.00002 e/a_0^3.

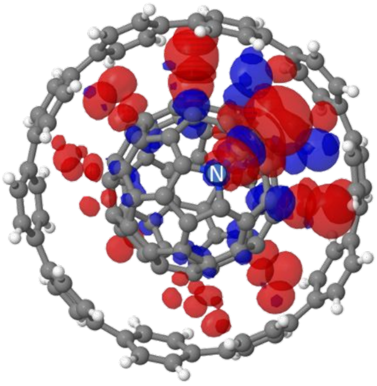

Figure S3. Subtraction of $C_{59}N^{\bullet}$ spin density from $C_{59}N^{\bullet}\subset$[10]CPP (**structure B**) spin density. Red and blue indicate positive and negative differential spin densities, respectively. The spin distributed in the blue region in $C_{59}N^{\bullet}$ moves to the red region when the [10]CPP is added. Isosurface cut-off value is set at 0.002 e/a_0^3. Grey, white, and blue balls represent carbon, hydrogen, and nitrogen atoms, respectively.

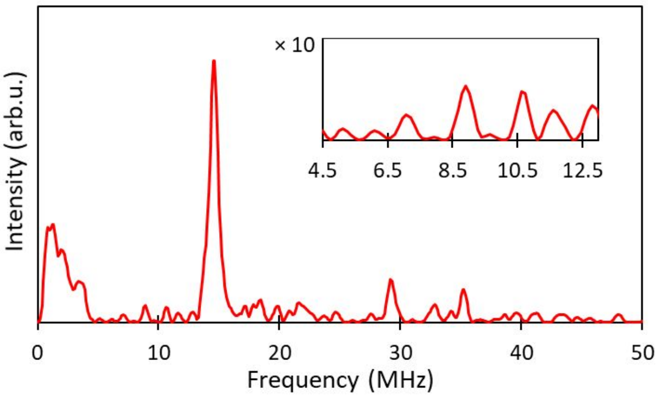

Figure S4. Fourier transformation of the raw experimental 2-pulse ESEEM spectrum. Inset shows enlarged spectrum between 4.5 and 12.5 MHz.

Table S1. Summary of difference in DFT-calculated parameters among $C_{59}N^{\bullet}$ without [10]CPP, $C_{59}N^{\bullet}\subset$[10]CPP complexes with N atom pointing toward CPP, and $C_{59}N^{\bullet}\subset$[10]CPP whose N sticks out of [10]CPP. A^{C} represents hyperfine coupling constant for the C atom on which the largest amount of the spin is distributed (radical C). Axes 1, 2, and 3 are orthogonal each other. Purple bubble plot shows unpaired electron distribution with a cut-off value of 0.002 e/a_0^3. Grey, white, and blue balls represent carbon, hydrogen, and nitrogen atoms, respectively.

	$C_{59}N^{\bullet}$ only	$C_{59}N^{\bullet}\subset$[10]CPP with N atom pointing toward [10]CPP (**structure A**)	$C_{59}N^{\bullet}\subset$[10]CPP with N atom pointing out of [10]CPP (**structure B**)
A^{N}_{x}, A^{N}_{y}, A^{N}_{z}	6.71, 6.79, 21.84	6.11, 6.20, 20.63	6.87, 6.96, 21.85
A^{N}_{iso} (MHz)	11.78	10.98	11.89
A^{C}_{x}, A^{C}_{y}, A^{C}_{z}	16.69, 17.04, 88.47	16.21, 16.56, 86.73	16.09, 16.43, 87.44
A^{C}_{iso} (MHz)	40.73	39.83	39.99
g_1, g_2, g_3	2.0007, 2.0009, 2.0019	2.0007, 2.0009, 2.0017	2.0006, 2.0009, 2.0019
g_{iso}	2.0011	2.0011	2.0011
Spin population on CPP	-	0.50%	0.71%
Structure	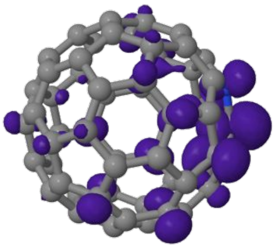	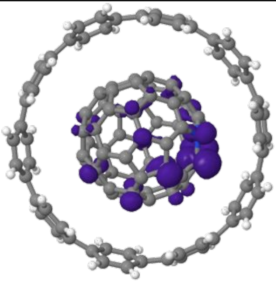	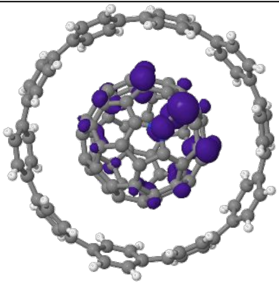

Table S2. Summary of A^{H}_{iso} values for **structure B** with absolute values larger than 0.1 MHz in [10]CPP and its C-H distance (r) from the radical carbon atom of $C_{59}N^{\bullet}$, calculated by DFT. Pink dashed arrows in the molecular structure in the right panel shows r with H label in the table.

H label	A_{iso} (DFT) [MHz]	*r* (DFT) [Å]
1	0.108	9.34
2	0.112	9.31
3	0.302	5.01
4	0.109	6.99
5	0.123	6.95
6	0.301	4.96

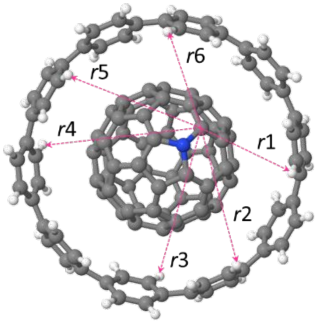

Annex S1. [10]CPP ring distortion and lp-π interaction

In addition to the dihedral angles of phenyl rings, the whole ring shape of [10]CPP also slightly deforms upon $C_{59}N^{\bullet}$ encapsulation. The ring diameter is measured at each inter-phenyl C atom to the point-symmetric opposite inter-phenyl C atom. Measured ring diameter of the isolated [10]CPP is 13.85 Å, without ring distortion (standard deviation is 0.005). While the [10]CPP ring with $C_{59}N^{\bullet}$ shows average diameter of 13.84 Å with standard deviation of 0.03, which varies from 13.80 Å (vicinity of the radical C) to 13.89 Å (≈90° rotated position from the radical C), suggesting there is additional weak interaction beside the π-π interaction close to the N atom of the $C_{59}N^{\bullet}$. One of possibility of this interaction may be the lone pair (lp)-π interaction[34] between the phenyl ring and the lone pair of the N atom. This may also explain that in the Ref. 8 experimentally the large part of $C_{59}N^{\bullet}\subset$[10]CPP complexation requires additional energy as thermal annealing. Our DFT calculations revealed that there is 0.1 e of the charge transfer from [10]CPP to the Au(111) surface before the deposition of $C_{59}N^{\bullet}$. The positively charged [10]CPP decreases its aromaticity and the decreased lp-π interaction results in a small energetic barrier for the complexation of $C_{59}N^{\bullet}\subset$[10]CPP. Once the system is thermally annealed after the deposition of $C_{59}N^{\bullet}$ on the [10]CPP, the [10]CPP ring starts moving towards $C_{59}N^{\bullet}$ energetically overcoming [10]CPP-Au interaction. The neutralised [10]CPP gains the lp-π interaction with the $C_{59}N^{\bullet}$ and this makes the $C_{59}N^{\bullet}\subset$[10]CPP complexation process barrierless.

Annex S2. Mathematical explanation of ESEEM modulation in the 2-pulse sequence.

For a system of coupled electronic S = 1/2 and nuclear spin I = 1/2, the modulation can be expressed as

$$V_{2\mathrm{p}}(\tau) = 1 - \frac{k}{4}\left[2 - 2\cos(\omega_\alpha\tau) - 2\cos(\omega_\beta\tau) + \cos(\omega_-\tau) + \cos(\omega_+\tau)\right]. \quad (Eq.\,\mathrm{S1})$$

Here, ω_α and ω_β are nuclear frequencies[35]

$$\omega_\alpha, \omega_\beta = \left[\left(\omega_\mathrm{L} \pm \frac{A_\perp}{2}\right)^2 \sin^2\theta + \left(\omega_\mathrm{L} \pm \frac{A_\parallel}{2}\right)^2 \cos^2\theta\right]^{\frac{1}{2}} \quad (Eq.\mathrm{S2})$$

determined by the nuclear Larmor frequency ω_L and parallel ($A_\parallel$) and perpendicular ($A_\perp$) components of hyperfine coupling tensor. θ is the angle between the external magnetic field and the eigen axis of the hyperfine coupling tensor. Another important parameter for the ESEEM experiments is so-called ESEEM modulation depth k, written as

$$k = \left(\frac{B\omega_\mathrm{L}}{\omega_\alpha\omega_\beta}\right)^2, \qquad (Eq.\mathrm{S3})$$

where B = $(A_\parallel - A_\perp)\sin\theta\cos\theta$.

Annex S3. Calculation of experimental hyperfine coupling constants from 2D plot of 3-pulse ESEEM.

According to Ref. 35, ω_α and ω_β are written as

$$\omega_{\alpha,\beta} = \left[\left(\omega_{\mathrm{L}} \pm \frac{A}{2}\right)^2 + \frac{B^2}{4}\right]^{\frac{1}{2}}, \quad (Eq.S4)$$

where

$$A = A_{\parallel}\cos^2\theta - A_{\perp}\sin^2\theta \; = T(3\cos^2\theta - 1) = \frac{1}{2}A_{\parallel} + \frac{1}{2}A_{\perp} \quad (Eq.\,S5)$$

$$B = (A_{\parallel} - A_{\perp})\,\sin\theta\cos\theta = 3T\sin\theta\cos\theta = 0, \quad (Eq.S6)$$

assuming that θ is averaged in the powder sample. *T* is dipolar coupling constant. Also

$$\omega_{+,-} = \left(\omega_{\mathrm{L}} + \frac{A}{2}\right)\cos\eta_\alpha \pm \left(\omega_{\mathrm{L}} - \frac{A}{2}\right)\cos\eta_\beta, \quad (Eq.S7)$$

where

$$\eta_{\alpha,\beta} = \arctan\left(\frac{-B}{A \pm 2\omega_{\mathrm{L}}}\right) = 0. \quad (Eq.\,S8)$$

Therefore from *Eqs.* S4-8, when hyperfine coupling *A* is isotropic,

$$\omega_{\alpha,\beta} = \left|\omega_{\mathrm{L}} \pm \frac{A}{2}\right|, \quad (Eq.S9)$$

$$\omega_{+,-} = \left(\omega_{\mathrm{L}} + \frac{A}{2}\right) \pm \left(\omega_{\mathrm{L}} - \frac{A}{2}\right). \quad (Eq.S10)$$

We derive the ^1H hyperfine constant from ω_α = 14.6 MHz as follows;

$$\omega_\alpha = 14.6 = \left|14.53 + \frac{A}{2}\right|$$

A = 0.14 MHz

In the same way, by using ω_α = 11.2 MHz for ^{13}C with an assumption of $A < 0$,

$$\omega_\alpha = 11.2 = \left|3.65 + \frac{A}{2}\right|$$

A = -29.7 MHz

For ^{14}N, by using ω_β = 3.4 with an assumption of $A > 0$

$$\omega_\beta = 3.4 = \left|1.05 - \frac{A}{2}\right|$$

A = 8.9 MHz
